# TEAM BASED LEARNING, AN OPPORTUNITY TO PROMOTE INTERPROFESSIONALISM IN GERIATRICS EDUCATION: A SYSTEMATIC REVIEW

**DOI:** 10.1093/geroni/igad104.3619

**Published:** 2023-12-21

**Authors:** Renzo Pajuelo-Vasquez, Wendy Gutierrez-Baca, Javier Flores-Cohaila, Fernando M Runzer-Colmenares

**Affiliations:** Universidad Científica del Sur, Lima, Lima, Peru; Universidad Científica del Sur, Lima, Lima, Peru; Universidad Científica del Sur, Lima, Lima, Peru; Universidad Científica del Sur, Lima, Lima, Peru

## Abstract

Geriatric care is complex and requires high coordination between healthcare providers. Thus, interprofessional education remains a priority, and innovative teaching methods are needed. Team-Based Learning (TBL) - “an active learning and small group instructional strategy that focuses on higher-cognitive abilities through a sequence of activities including individual work, teamwork and immediate feedback through significant problems.” – appears as one of the most valuable solutions. Following BEME recommendations, this systematic review examined the current evidence on TBL in health professions education (HPE). PubMed, EMBASE, WoS, and Scopus were searched from inception till August 2023 using key terms/subject headings related to TBL and Geriatrics. Four studies published between 2013 and 2022 met our inclusion criteria. Two studies included solely nurses and the remaining two – students from different disciplines. One study was quasi-experimental, while others were cross-sectional. Studies involved undergraduate (n=4) and graduate (n=2) students. Geriatric care was the curriculum’s focus in two studies, while it was a component in the remaining two (covered in one or two modules). All studies followed the sequence of a TBL session. Although TBL was not preferred over traditional lectures for students (n=1), there was a gain in knowledge and skills in most of the studies (n=3). To analyze the quality of studies, the MERSQI was employed (Mean = 9.87, SD =1.31), but there was a lack of validity in assessment instruments and zero patient-reported outcomes. TBL is an opportunity to promote interprofessional education. However, further studies using better assessment methods and higher-level outcomes are needed.

